# Mutation analysis reveals novel and known mutations in SAG gene in first two Egyptian families with Oguchi disease

**DOI:** 10.1186/s12886-022-02444-5

**Published:** 2022-05-12

**Authors:** Caroline Atef Tawfik, Nagham Maher Elbagoury, Noha Ibrahim Khater, Mona Lotfi Essawi

**Affiliations:** 1 Department of Ophthalmology, Ain Shams University, 38 Abbasseya, Nour Mosque, El-Mohamady, Al Waili, Cairo, 11566 Egypt; 2 Medical Molecular Genetics Department, Human Genetics and Genome Research Institute, National Research Centre, Cairo, Egypt; 3 Center of Excellence for Human Genetics, National Research Centre, Cairo, Egypt; 4 Department of Ophthalmology, Cairo University, Giza, Egypt; 5 Al Mouneer Diabetic Eye Center, Dokki, Giza, Egypt

**Keywords:** Africa, Egypt, Novel canonical splice site mutation, Oguchi disease, and *S-antigen (SAG)* gene

## Abstract

**Background:**

Oguchi disease is a rare type of congenital stationary night blindness associated with an abnormal fundus appearance. It is inherited in an autosomal recessive manner where two types exist according to the gene affected; type 1 associated with S-antigen *(SAG)* gene mutations and type 2 associated with rhodopsin kinase *(GRK1)* gene mutations.

**Purpose:**

The aim of this work was to describe the clinical and genetic findings of the first two reported families of Oguchi disease in Egypt and African region.

**Methods:**

Four members of two consanguineous Egyptian families with history of night blindness since childhood underwent complete ophthalmological examination, standard automated static perimetry, fundus color photography, fundus autofluorescence (FAF), fundus fluorescein angiography (FFA) in light-adapted state and spectral-domain optical coherence tomography (SD-OCT) of both the macula and the optic nerve head as well as central corneal thickness with repeated fundus photography following prolonged dark adaptation. Mutation screening of 7 coding exons of *GRK1* gene and 15 coding exons of *SAG* gene as well as some flanking regions were performed using Sanger sequencing technique. The variants were tested for pathogenicity using different in silico functional analysis tools.

**Results:**

The clinical examination and investigations confirmed Oguchi disease phenotype. One patient showed p.R193* (c.577C > T) which is a previously reported *SAG* gene mutation in a homozygous form. The other three patients from a different family showed (c.649–1 G > C), a novel canonical splice site *SAG* gene mutation in a homozygous form.

**Conclusion:**

The identification of the novel canonical splice site *SAG* gene variant in three members of the same family with clinically confirmed Oguchi disease reinforces its pathogenicity. A fourth patient from another family carried a previously reported mutation in the same gene. *SAG* gene variants may be the underlying genetic cause for Oguchi disease in Egypt. Our findings have expanded the spectrum of Oguchi disease-associated mutations in *SAG* gene and may serve as a basis for genetic diagnosis for Oguchi disease.

## Background

Oguchi disease is considered one of the rare forms of congenital stationary night blindness (CSNB) that is inherited in an autosomal recessive manner. It is classified into two types: type 1 and type 2, each related to separate genetic background. Mutations in arrestin gene (*SAG*, S-antigen; OMIM:181031) were first discovered in Japanese patients (Oguchi disease type 1) [1], later on, mutations involving the rhodopsin kinase gene (*GRK1*; OMIM:180381) were implicated in European patients (Oguchi disease type 2) [2]. Both genes encode proteins involved in recovery of rhodopsin after photoactivation [1].

*GRK1* gene is mapped to chromosome 13. It is a specialized G-protein-coupled receptor kinase that eventually results in phototransduction cascade shutdown [3, 4]. Various mutations have been reported in this gene, which have been shown to result in a decline in the catalytic activity of the protein, leading to a delay in photoreceptor recovery [5, 6].

*SAG* gene is located on chromosome 2, consists of 16 exons of which 15 are coding, and it encodes for arrestin which is a 405 amino acid regulatory cytosolic protein. Arrestin forms a complex with photoactive rhodopsin, thereby uncoupling it from the G-protein transducin, and thus playing a critical role in recovery phase of phototransduction [3, 7]. Pathogenic variants detected in *SAG* gene can manifest as Oguchi inherited in autosomal recessive manner as well as retinitis pigmentosa (RP) inherited in autosomal dominant manner. To date, around 18 pathogenic variants have been detected in the gene including 1 gross insertion, 2 gross deletions, 2 small deletions and 13 missense/nonsense mutations [8].

Clinically, Oguchi disease is characterized by non-progressive retinal dysfunction as well as a unique golden-yellow fundus with a metallic sheen that reverts to normal fundus color after prolonged dark adaptation. It was first reported by Oguchi in 1907 as a variant of CSNB. Later, it was further phenotypically characterized by Mizuo in 1913 by demonstrating the Mizuo-Nakamura phenomenon where the fundus discoloration disappears, leaving normal fundus appearance after 3–4 h of dark adaptation, only to reappear again shortly after exposure to light [9].

Oguchi disease has been demonstrated to be more prevalent among the Japanese population as compared to other populations [1]. To the authors’ knowledge, around 50 cases of Oguchi disease have been reported worldwide and those have been primarily concentrated in Japan with few reported cases from Europe [10–13], Turkey [14], China [15], India [16], Pakistan [17, 18] and Iran [19].

Oguchi disease has not been described so far in the Egyptian population or in any African population, and the mutation spectrum remains unknown whether *SAG* or *GRK1* mutations will prevail. Thus, we set out this study to report and characterize the first cases encountered both clinically and genetically.

## Methods

Four members of two indigenous Egyptian families with history of night blindness were examined at a general comprehensive clinic of Al Mouneer Diabetic Eye Center, Cairo, Egypt.

The enrolled patients underwent a complete ophthalmological examination that included recording of medical, ocular and family histories, unaided distance visual acuity (UDVA) and corrected distance visual acuity (CDVA) testing using the Snellen chart, subjective refraction, color vision testing by Ishihara chart (38-plate edition), ocular motility examination, intraocular pressure measurements using Goldmann applanation tonometry, as well as slit-lamp biomicroscopy and a dilated fundus examination. All patients refrained from undergoing electrophysiological studies as they were unavailable in our center and had to be performed elsewhere.

Standard automated static perimetry (Vision Monitor, Metrovision, Pérenchies, France) was done for both eyes of each patient pre-dilation of the pupils. Fundus color photography, fundus autofluorescence (FAF) and fundus fluorescein angiograms (FFA) were done in light-adapted state post-dilation of the pupils. In order to confirm the diagnosis, patients were dark adapted for 3–4 h following which the fundi were re-imaged, and spectral-domain optical coherence tomography (SD-OCT) of both the macula and the optic nerve head as well as central corneal thickness were performed with Topcon 3D OCT-2000FA plus (Oakland, NJ, USA).

This study adhered to the tenets of the Declaration of Helsinki and the study protocol was approved by the International Review Board of Watany Eye Hospitals, Cairo, Egypt (registration code RET-2020–002), and informed consents were obtained from all patients after providing an explanation of the purpose of this study.

### Mutation screening

Genomic DNA was extracted from peripheral blood leukocytes using the salting out protocol [20]. The 7 coding exons of *GRK1* gene (NM_002929.3), the 15 coding exons of *SAG* gene (NM_000541.5) as well as a flanking intronic sequences of at least 50 bp have been amplified to detect any splice site variant using specially designed primers by primer3 software. Table 1 shows the primers for coding exons of *SAG* gene. Successfully amplified fragments were purified using Exo-SAP PCR kit (Fermentas, Germany) followed by sequencing in both directions using the BigDye Terminator v3.1 Cycle Sequencing Kit (Applied Biosystems, Foster City, CA) and analyzed on the ABI Prism 3500 Genetic Analyzer (Applied Biosystems) according to instructions supplied by manufacturer. Obtained sequencing data was aligned against reference genomic and complementary DNA of each gene. Detected variants were checked in publicly available control databases followed by check for pathogenicity using in silico pathogenicity prediction tools as MutationTaster (http://www.mutationtaster.org), NNSplice (http://www.fruitfly.org/seq_tools/splice.html), NetGene2 (http://www.cbs.dtu.dk/services/NetGene2/), GENSCAN (http://genes.mit.edu/GENSCAN.html), MaxEntScan (http://genes.mit.edu/burgelab/maxent/Xmaxentscan_scoreseq.html), and Spliceman (http://fairbrother.biomed.brown.edu/spliceman/index.cgi).

**Table 1:** Primers for coding exons of SAG gene used in PCR

Exon	Primer	Fragment Size (bp)
2	F: GGATGTTCTTGACTTGATATTTAGGA R: ATGTTGATCATGACTACACTGTACTTG	265
3	F: GGCTGCTGCTGGTTTTTATC R: CACAAAGTGAGCGGTTATCTG	206
4	F: TTCCCTTTGCCTGACTTTTC R: GCCCAGAGAGCAGTCCAC	245
5	F: GGCTGATTCTCCAGAGTGGT R: CTTTCAAGGGTGCAATTTCC	495
6	F: TCAGGAGTATAGGTGAATGTGATTTT R: CCCAGCATTGGTGACAGAGT	300
7	F: TGCTTCCAAATCCCTAAATG R: CTTTGACCTCCATCCCACAT	382
8	F: CTCAGGCTCTGACCAGTGT R: CACTTACGCATGTTGGTGCT	442
9	F: TGTTTCAGGCCCTTCCTTAG R: CAGTCCTGCTGCCTATGACA	389
10	F: TGAGAAAGCCTAGCCTTGGA R: AGGGGTGTGGTAGATGCAGA	389
11	F: AGGCTCTTGAACCCACCTTC R: GTGTAACCCCCACATCACAC	467
12	F: AGGACTTTGGAAGCTCAGTG R: CCCAGTCATTCAGGAAAGGA	300
13	F: GGAAGACCCTGGATGTTGTG R: GGCAAAGCATCATTCCTAACA	294
14	F: AGAATTGCATGAACCCAGGA R: TGAGATGCGGTCAAGAAAGA	385
15	F: GCAGGAATTACACGCAGTGA R: TTTGTGCTGGAGGAGAAGGT	248
16	F: GTGCCCCCATGTTCTATCA R: CTTTGCTCGTTGCACTGGTA	290

*Bp* Base pair, *F* Forward, *R* Reverse

## Results

We examined four patients of two unrelated families, where both families gave history of consanguinity. Table 2 shows the results of our collected data.

**Table 2:** Age, Sex, Visual findings, and Mutation in Studied Patients^a^

Patient	Eye	Age/Sex	Night Blindness	CDVA	Refraction	Mizuo-Nakamura	SD-OCT	Mutation
1	*OD OS*	55/F	Stationary since childhood	20/20 20/20	+ 2.25 + 1.75	Present	Indiscernible outer segments of PRs in the parafovea	P.R193^a^ (c.577C > T)
2	OD OS	65/M	Stationary since childhood	20/25 20/25	Pseudophakia, tessellated fundus	Present	Indiscernible outer segments of PRs in the parafovea	Canonical splice site (c.649–1 G > C)
3	OD OS	31/M	Stationary since childhood	20/20 20/20	Emmetropia	Present	Indiscernible outer segments of PRs in the parafovea	Canonical splice site (c.649–1 G > C)
4	OD OS	28/M	Stationary since childhood	20/20 20/20	Emmetropia	ND	Indiscernible outer segments of PRs in the parafovea	Canonical splice site (c.649–1 G > C)

*CDVA* Corrected distance visual acuity, *F* Female, *M* Male, *ND* Not done, *OD* Right eye, *OS* Left eye, *PRs* Photoreceptors, *SD-OCT* Spectral-domain optical coherence tomography

^a^ Detailed results of patients’ examination

### Clinical findings

Our first patient (patient 1) was a 55-year-old female patient who was coming for a baseline checkup for diabetic retinopathy following her new diagnosis of type 2 Diabetes mellitus (DM) a month earlier. She was aware of night blindness dating since childhood which was diagnosed as vitamin A deficiency for which she received medical treatment with no improvement. Otherwise, her ocular history was unremarkable. There was no history of night blindness in either the mother or the father. The patient was unsure about her grandparents ever complaining of night blindness.

**Fig. 1:**
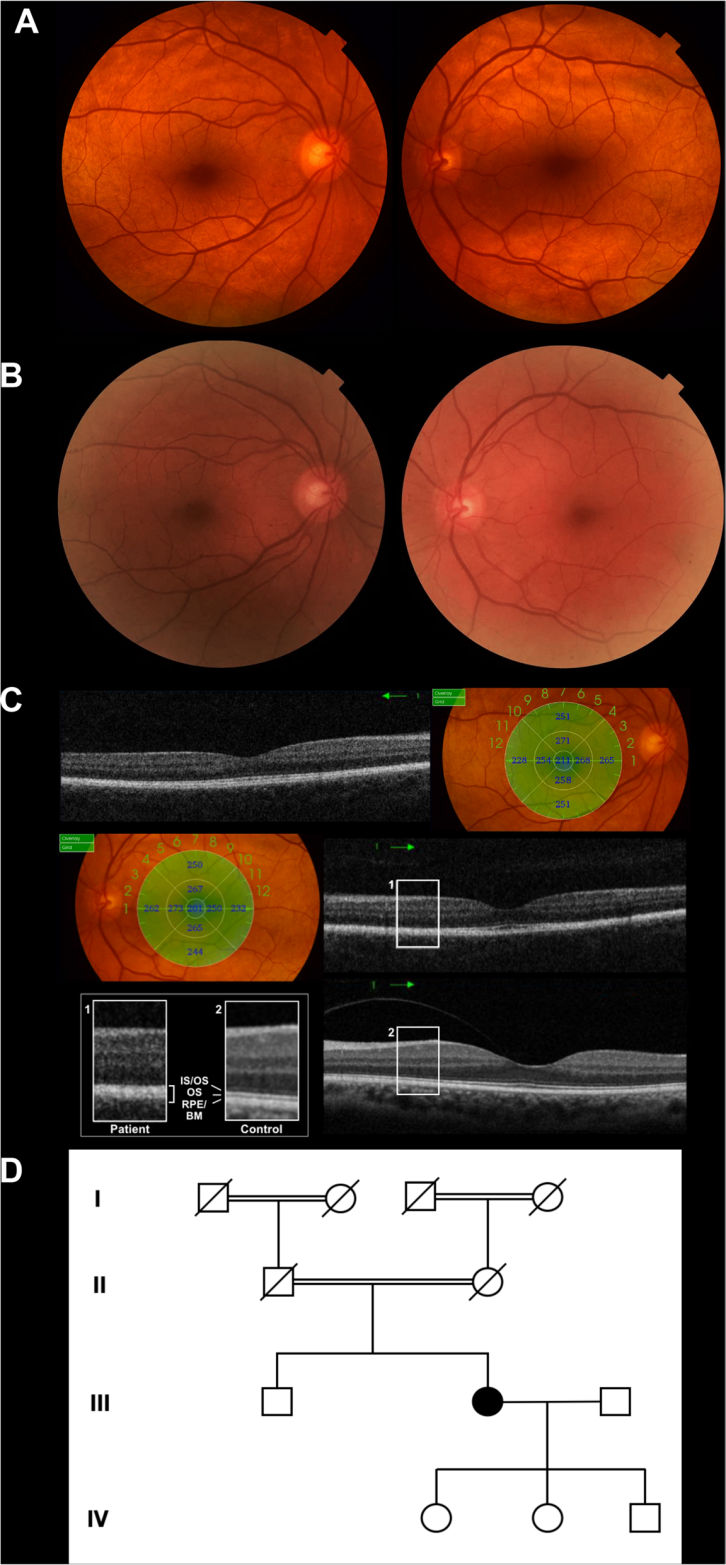
Clinical and genetic data of the 55-year-old patient from the first family (patient 1). **A** Color fundus photograph of the right and left eye showing the characteristic golden-yellow reflex involving the macula. **B** Mizuo-Nakamura phenomenon is seen as the fundus color of both eyes reverts back to normal following prolonged dark adaptation. **C** Spectral domain optical coherence tomography of the right and left eyes showing indiscernible hyporeflective band of outer segments of photoreceptors between the hyper-reflective layers associated with the ellipsoid zone, interdigitation zone and the densely packed RPE/Bruch’s membrane complex in the parafoveal region. Enlarged images of boxed regions (1, patient, 2, control) shows the outer retina in detail. **D** Four-generation family pedigree highlighting the patient

Her medical history was unremarkable, except for the recent diagnosis of type 2 DM. CDVA was 20/20 bilaterally with a hyperopic refraction of + 2.25 D sphere, -1.5 D cylinder in the right eye, and + 1.75 D sphere, -0.25 D cylinder in the left eye. The Mizuo-Nakamura phenomenon was demonstrated after three hours of dark adaptation. No changes were identified on both FAF and FFA. Central corneal thickness was 575 µm in the right eye, and 579 µm in the left eye. SD-OCT was unable to detect the hyporeflective band corresponding to outer segment of photoreceptors between the hyper-reflective layers associated with the ellipsoid zone (EZ), the interdigitation zone (IZ) and a densely packed retinal pigment epithelium (RPE)/Bruch's membrane complex in the parafoveal region as shown in Fig. 1.

The other highly consanguineous family had three members; a 65-year-old male patient (patient 2) along with his two sons (patient 3 and 4), who presented for a routine checkup. The father was medically-free, with past ocular history significant for bilateral phacoemulsification with posterior chamber intraocular implants done 2 years prior to the current date. When questioned about night blindness, he assented that he had it all his life. Moreover, he confirmed that his late mother as well as both his sons suffered from the same condition.

CDVA was 20/25 bilaterally. Anterior segment examination showed well-centered posterior chamber intraocular implants. Upon presentation, his IOP was found to be elevated with pressures of 36 mmHg bilaterally. His fundus examination revealed the characteristic golden-yellow reflex in all areas of the fundus. The Mizuo-Nakamura phenomenon was demonstrated and documented. FAF and FFA imaging were normal. Central corneal thickness was 599 µm in the right eye, and 613 µm in the left eye.

**Fig. 2:**
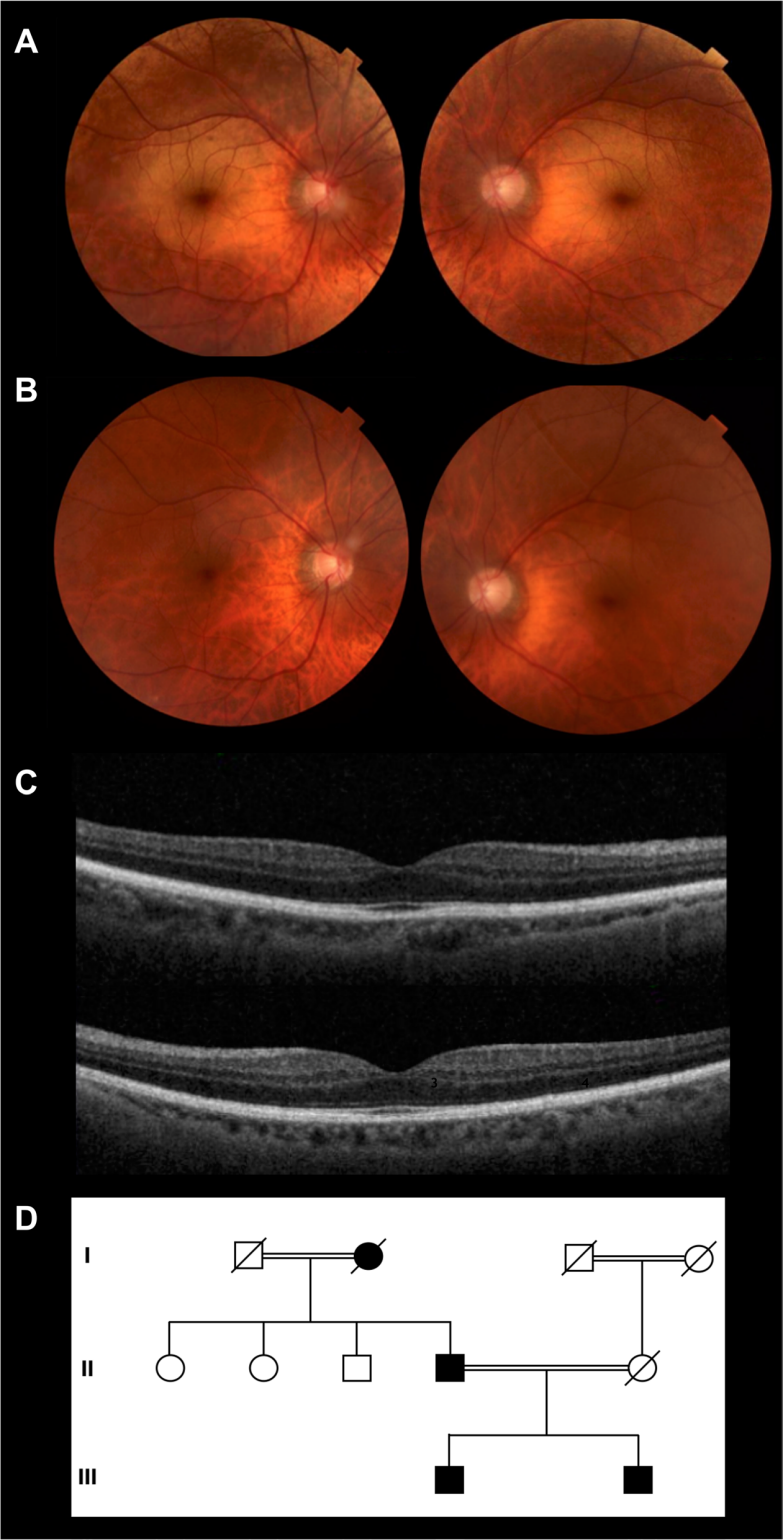
Clinical and genetic data of the 65-year-old patient from the second family (patient 2). **A** Color fundus photograph of the right and left eye showing the characteristic golden-yellow reflex involving the macula. **B** Mizuo-Nakamura phenomenon is seen as the fundus color reverts back to normal following prolonged dark adaptation. **C** Spectral domain optical coherence tomography of macula of right and left eyes showing indiscernible outer segments of photoreceptors. **D** Three-generation family pedigree highlighting the patient and other affected family members; note the consanguinity in the two generations on both sides of the family

SD-OCT of the macula showed the same finding of an indiscernible hyporeflective layer of outer segment of photoreceptors between the hyper-reflective layers associated with the EZ, IZ and a densely packed RPE/Bruch's membrane complex in the parafoveal region as demonstrated in the first patient as shown in Fig. 2.

**Fig. 3:**
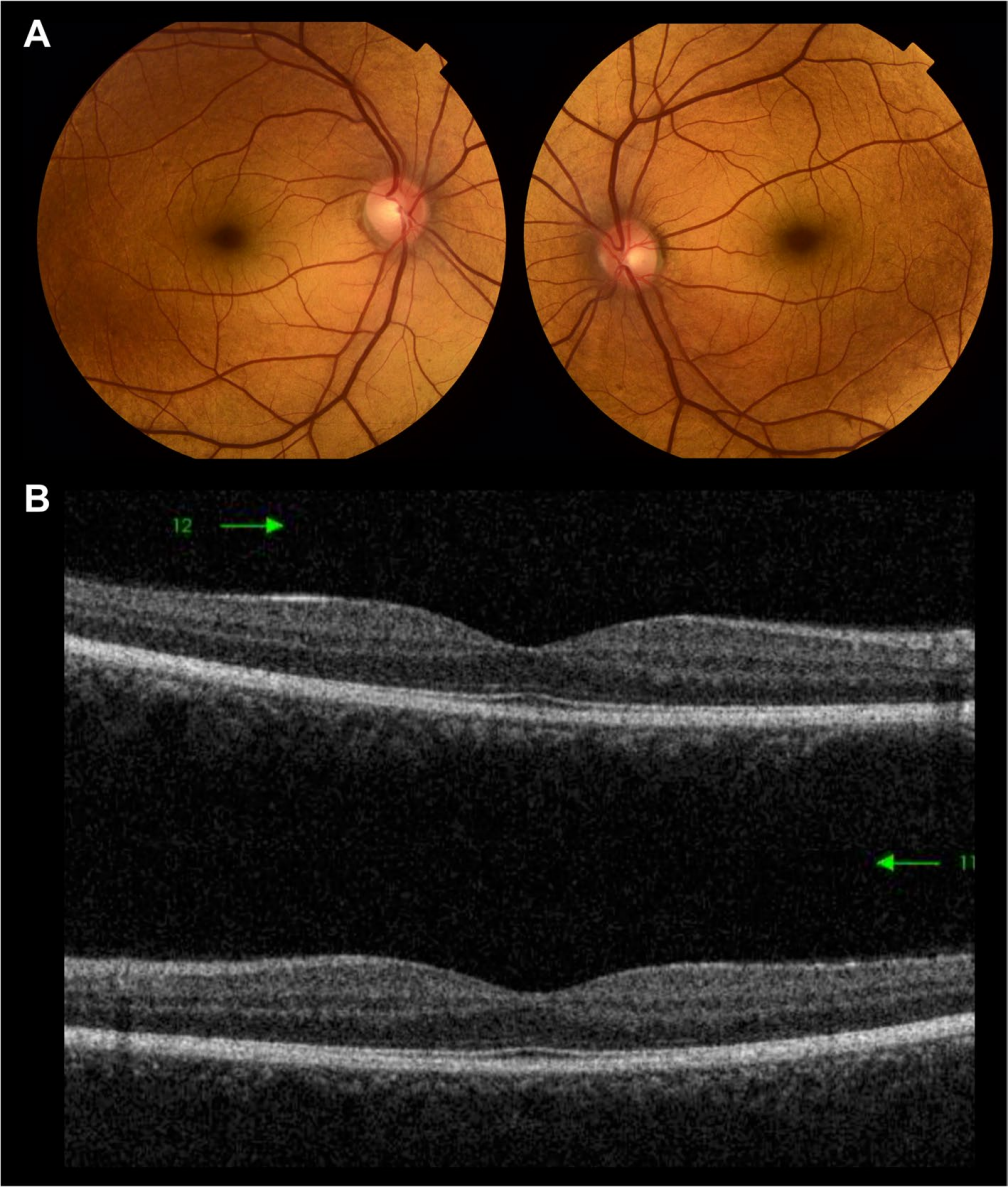
Clinical images of the 31-year-old patient 3. **A** Color fundus photograph of the right and left eye showing the characteristic metallic reflex involving the macula. **B** Spectral domain optical coherence tomography of macula of right and left eyes showing indiscernible outer segment of photoreceptors

**Fig. 4:**
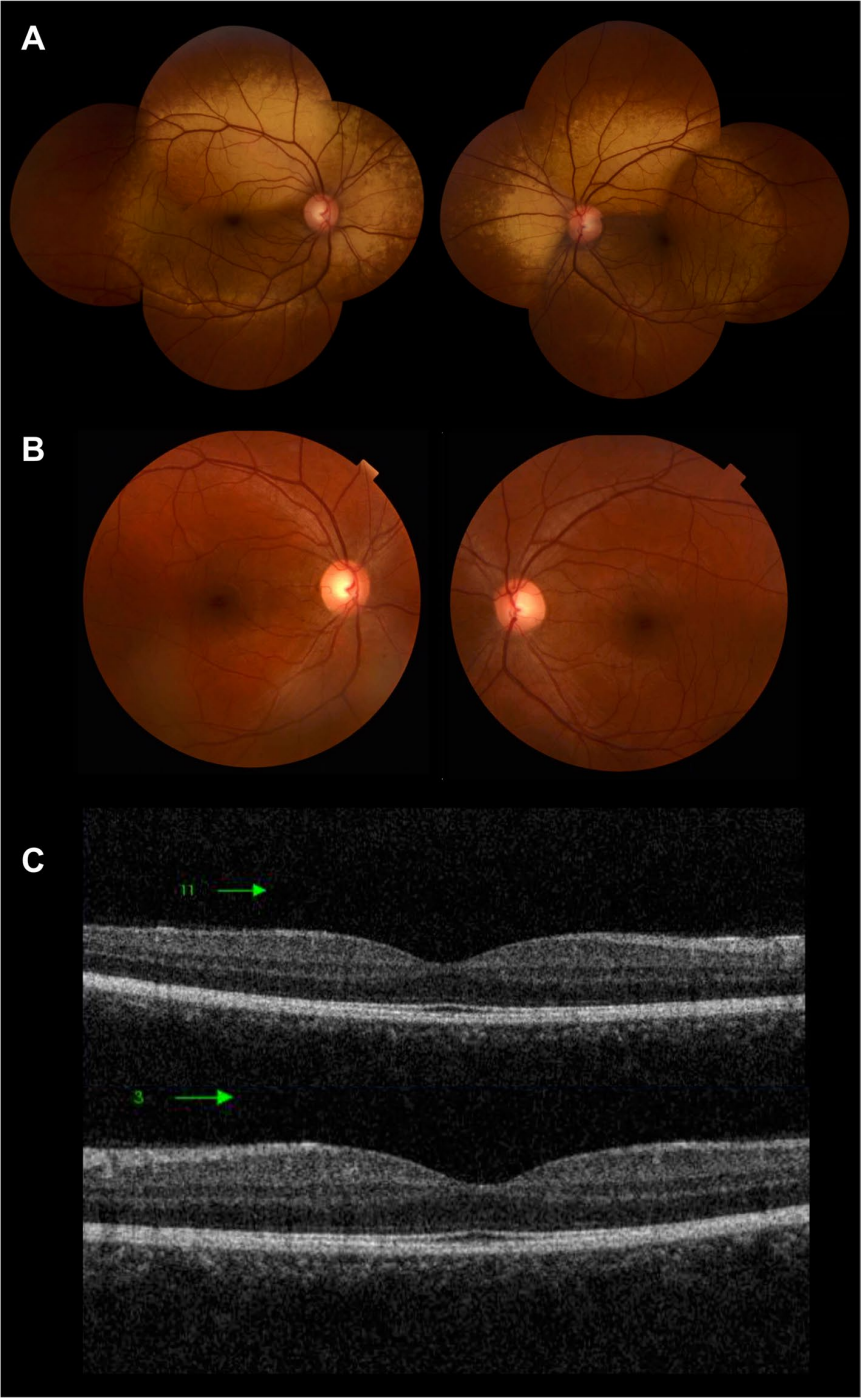
Clinical images of the 28-year-old patient 4. **A** Color fundus photograph of the right and left eye showing the characteristic metallic reflex involving the macula, extending beyond the vascular arcades. **B** Mizuo-Nakamura phenomenon is seen as the fundus color reverts back to normal following prolonged dark adaptation. **C** Spectral domain optical coherence tomography of macula of right and left eyes showing indiscernible outer segment of photoreceptors

Both of his sons were recruited for complete ophthalmological examination, the elder was a 31-year-old (patient 3) and the younger (patient 4) was a 28-year old. They both suffered from stationary night blindness since childhood and consented to giving blood samples for genetic testing. UDVA was 20/20 bilaterally. Anterior segment examination was within normal limits. Examination revealed within normal intraocular pressures. Fundus examination revealed the characteristic golden-metallic sheen. The Mizuo-Nakamura phenomenon was ascertained and documented in the younger son, while the elder patient refrained from undergoing prolonged dark adaptation. All the investigations were within normal limits except for the same finding of indiscernible outer segment of photoreceptors on SD-OCT as shown in Figs. 3 and 4 respectively.

### Molecular genetic study

Sequencing of the seven exons of the *GRK1* gene disclosed no sequence changes for any of the four patients. On the other hand, Sanger sequencing for the 15 coding exons of *SAG* gene showed two pathogenic variants. Patient 1 had the previously reported nonsense mutation p.R193* (c.577C > T) in a homozygous form, whereas patients 2, 3 and 4 from the same family had a novel canonical splice site mutation in a homozygous form (Chr2:g.234238138G > C (hg19); NM_000541.5; c.649–1 G > C) with accession assigned by ClinVar (SCV001451934.1). The novel variant wasn’t previously present in publicly available control databases as 1000 genomes, NHLBI exome sequencing project or gnomAD. Performed in silico functional analysis favored a pathogenic effect for the novel splice site mutation as shown in Table 3.

**Table 3:** In silico analysis for the novel splice site mutation c.649–1 G > C (SCV001451934.1) ^a^

Tool	Output	Interpretation	Score of c. 649–1 G > C
NNSplice	Score (0–1)	Higher score increases the probability of affecting the splice site	0.96
NetGene2	Confidence score (0–1)	Higher confidence score indicates higher confidence of true splice site	0.96
GENSCAN	I/Ac initiation signal or acceptor splice site score (× 10)	If below zero, probably not a real acceptor site	0.998
Mutation Taster	Probability score (0–1)	Higher score predicts higher pathogenic effect	1
Spliceman	L1 distance and percentile rank	Higher percentile rank indicates higher ability to disrupt splicing	L1 distance = 34,421 Percentile rank = 64%
MaxEntScan	Maximum entropy score (log odds ratio)	Higher score indicates a higher possibility of the sequence being a true splice site	-4.45

*Ss* Splice site

^a^ Summary of output, and interpretation of prediction scores for in silico tools used along with ClinVar accession number

## Discussion

Oguchi disease is an exceedingly rare autosomal recessive disease. It was first described in Japanese subjects and is characterized by Mizuo-Nakamura phenomenon as well as abnormally slow dark adaptation manifesting as night blindness. Other visual functions, such as visual acuity, visual field, color vision and daytime vision are usually within normal [1]. The disease has two forms: Oguchi type 1 (OMIM 258100) caused by mutations in the *SAG* gene (OMIM 181031) and Oguchi type 2 (OMIM 613411) caused by mutations in the *GRK1* gene (OMIM 181031) [21].

It has been suggested that Oguchi type 1 and 2 diseases are liable to ethnic-specific or geographical distribution [14]. Indeed, *SAG* mutations are concentrated in Japanese patients of Oguchi disease while *GRK1* mutations are very rarely reported in Japanese patients [22]. Compound heterozygous variations in the *SAG* gene were detected in a Chinese family clinically diagnosed as Oguchi disease [15], whereas another showed homozygous variation [21]. On the other hand, *GRK1* gene mutations are mostly found in southern Asians and Europeans patients with Oguchi disease, with very few reports of families harboring *SAG* gene mutations [16, 17, 23]. No studies reported any patients with Oguchi disease in Egyptian or African population, and it remained unclear whether they harbor SAG gene or GRK1 gene mutations.

Moreover, there were reports of slowly progressive visual dysfunction in *SAG*-associated Oguchi disease (type 1) secondary to minimally progressive retinal degeneration, which resulted in some cases of the disease being re-classified as RP many years after initial diagnosis with Oguchi disease [24, 25]. Hence, the importance of identifying the exact genotype as it may imply life-long follow up of patients for fear of possibility of RP development.

Normally, in the fovea, three hyper-reflective bands representing the EZ, the IZ, and the RPE/Bruch’s membrane complex are visible. However, several studies [3, 7, 26] reported that only two distinct lines were discernible in the parafoveal area on SD-OCT findings in Oguchi disease suggesting that microarchitectural changes may occur in the photoreceptors. The densely packed structure in the parafovea was observed in all of our four patients which may suggest that it is a rather constant feature of Oguchi disease.

Among the mutations related to Oguchi disease in SAG gene are; a gross deletion of 3224 bp including exon 2 [15], a small deletion of one nucleotide in codon 309 [1], and around 5 missense/nonsense mutations. These include one mutation in exon 5 p.96Lfs*28 [21], two mutations in exon 8 p.Arg175* [27], p.Arg193* [16], and two mutations in exon 11 p.Arg292* and p.Glu306* [17].

In our study, molecular screening of the 7 coding exons of the *GRK1* gene revealed normal sequence excluding Oguchi disease type 2. On carrying molecular screening of the 15 coding exons of the *SAG* gene, patient 1 showed the previously reported nonsense mutation p.R193* (c.577C > T) in a homozygous form. Production of a truncated arrestin protein of 193 amino acid instead of the 405 amino acid would lead to configuration problems.

Upon carrying the mutation analysis of patients 2, 3 and 4 (the father and his two sons), all patients carried a novel canonical splice site mutation at the acceptor region of intron 9. The mutation was one base before exon 9 (c.649–1 G > C) in a highly conserved region. We couldn’t obtain a blood sample from a healthy member of the family for segregation analysis as the mother was deceased and there were no grandchildren to date. On performing in silico functional analysis using different tools, they all favored the pathogenic effect of the novel canonical splice site mutation affecting the intronic AG region as shown in Table 2. The mutation variations that affect intronic GT‐AG dinucleotides compromise pre‐mRNA splicing [28]. Functional protein production requires accurate splicing of pre‐mRNA otherwise hereditary disorders might be a consequence [29]. Correct splicing is affected by the process of recognition and selection of the proper donor and acceptor splice sites which define the introns’ borders [30].

In our study, the novel splice site mutation was encountered in the 3’ AG acceptor region of intron 9 just before exon 9 which lies within the C-terminal domain of arrestin [31]. C-domain is responsible for membrane engagement and stabilization of the formed arrestin-receptor complex through its way to tight binding. It is also involved in the binding specificity since the activation of the C-domain doesn’t occur unless arrestin is activated by the phosphorylated receptor C terminus [32]. c.649–1 G > C splice site mutation is predicted to hinder the proper transcription of the gene and hence the proper production of normal mRNA which might be subjected to nonsense mediated mRNA decay or end up in an aberrant nonfunctioning protein. This splice site variant is the third to be found in *SAG* gene as the first splice site mutation in *SAG* gene was described by Liu et al. in a Chinese Oguchi patient in a homozygous form [21] and the second one was described in another Chinese Oguchi patient in a compound heterozygous form [33].

Mutations in the non-coding region of *SAG* gene may play a role in the pathogenesis of Oguchi disease, so should be considered in genetic testing of Oguchi disease patients. Furthermore, identification of additional families with the same mutation would enable better understanding of genotype–phenotype correlation.

## Conclusion

To the best of our knowledge, this is the first study documenting cases of Oguchi disease in Egypt and in whole Africa. *SAG* gene mutations were confirmed in all four of our enrolled patients diagnosed with Oguchi disease. The novel splice site mutation (c.649–1 G > C) is the third splice site mutation to be detected in the *SAG* gene so far. Our findings have expanded the spectrum of Oguchi disease associated mutations in *SAG* gene and may serve as a basis for genetic diagnosis for Oguchi disease.

## Data Availability

All data generated or analyzed during this study are included in this article. Further enquiries can be directed to the corresponding author.

## Abbreviations

SAG: S-antigen
GRK1: Rhodopsin kinase
FAF: Fundus autofluorescence
FFA: Fundus fluorescein angiography
SD-OCT: Spectral-domain optical coherence tomography
CSNB: Congenital stationary night blindness
RP: Retinitis pigmentosa
UDVA: Unaided distance visual acuity
CDVA: Corrected distance visual acuity
PCR: Polymerase chain reaction
DM: Diabetes mellitus
EZ: Ellipsoid zone
IZ: Interdigitation zone
RP: Retinitis pigmentosa
RPE: Retinal pigment epithelium
